# Distinct role of central predictive mechanisms in tactile suppression

**DOI:** 10.1016/j.isci.2024.110582

**Published:** 2024-07-25

**Authors:** Belkis Ezgi Arikan, Dimitris Voudouris, Benjamin Straube, Katja Fiehler

**Affiliations:** 1 Department of Experimental Psychology, Justus Liebig University Giessen, Otto-Behaghel Strasse 10F, Philosophikum I, 35394 Giessen, Germany; 2 Center for Mind, Brain and Behavior (CMBB) of the University of Marburg, Justus Liebig University Giessen and University of Darmstadt, Hans-Meerwein-Strasse 6, 35032 Marburg, Germany; 3 Department of Psychiatry and Psychotherapy, University of Marburg; Rudolf-Bultmann-Strasse 8, 35039 Marburg, Germany

**Keywords:** Neuroscience, Behavioral neuroscience, Sensory neuroscience, Cognitive neuroscience

## Abstract

Tactile sensitivity on a limb is reduced during movement. This tactile suppression results presumably from central predictive mechanisms that downregulate sensations caused during voluntary action. Suppression also occurs during passive movements, indicating a role for peripheral mechanisms, questioning the predictive nature of suppression. Yet, predictions existing beyond the motor domain (non-motor predictions) can also modulate tactile suppression. This study aimed to disentangle central motor predictive and peripheral feedback mechanisms while accounting for non-motor predictions. Participants detected tactile stimuli on their limb shortly before it moved in an active or passive manner. Passive movements were either fully (100%) or partially (50%) predictable. We found tactile suppression during both active and passive movements irrespective of whether the passive movements were predictable. Importantly, tactile suppression was stronger in active than passive movements highlighting the specific role of central predictive mechanisms.

## Highlights

• Tactile sensitivity is reduced in both active and passive movements
• Tactile suppression is stronger for active than passive movements
• Results illustrate how motor predictions tune tactile sensitivity around movement

## Graphical abstract

**Figure:**
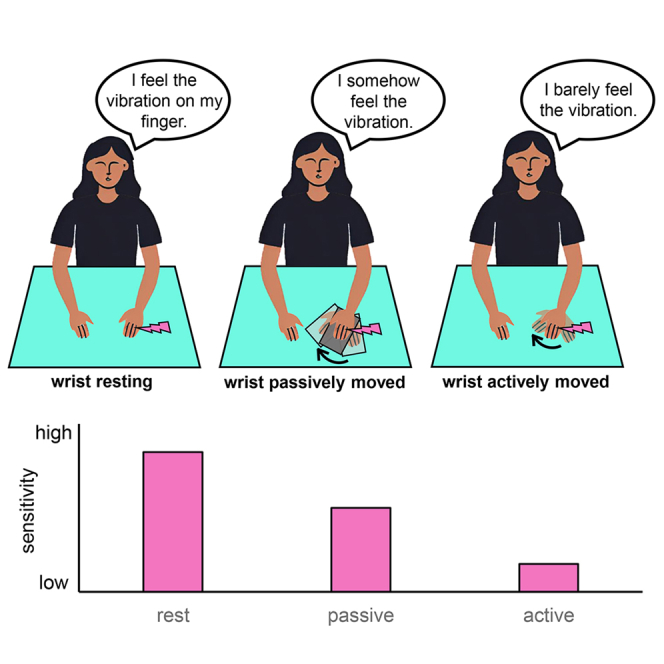

## Introduction

Our sense of touch is shaped by how we interact with our environment. During goal-directed movements, transmission of somatosensory signals to the primary somatosensory cortex is typically diminished.^1^^,^^2^^,^^3^ This is accompanied by decreased sensitivity to tactile stimuli on the moving limb, a phenomenon known as tactile suppression.^4^^,^^5^ Tactile suppression can be observed for sensations directly caused by voluntary movements (e.g., during self-touch),^6^^,^^7^^,^^8^ or for externally produced tactile stimuli that cause sensations on the moving limb (e.g., vibration on the moving finger).^9^^,^^10^^,^^11^^,^^12^ On the other hand, tactile sensitivity is enhanced during exploratory movements.^13^ These observations suggest that tactile suppression occurs for bodily signals as well as sensations that arise from our interactions with external stimuli, and that voluntary movements alter the sensitivity of a moving limb according to its relevance for the movement. It has been postulated that tactile suppression reflects a fundamental process that enables humans to distinguish between sensations arising from the movement from those occurring unexpectedly and to maintain a coherent sense of one’s own body and the external world.^6^^,^^14^ Through suppression, the nervous system can free up capacities to process unexpected changes, like obstacles or threats, more efficiently.^15^^,^^16^^,^^17^ The underlying mechanism is typically attributed to internal forward models that generate an efference copy of the motor command to predict and downregulate accompanying sensations from the movement.^18^^,^^19^^,^^20^ Accordingly, the cerebellar cortex uses internal forward models to decrease the transmission of sensory signals associated with the movement.^16^^,^^21^^,^^22^ This is supported by human and animal work demonstrating decreased somatosensory activity during limb movements at central cortical and spinal levels.^1^^,^^2^^,^^23^ Suppression begins shortly before movement onset, i.e., during movement preparation^11^^,^^16^^,^^17^ and is even evident when the movement is planned but not carried out, providing further support for the role of central motor predictions in regulating tactile sensitivity.^24^^,^^25^ Tactile suppression has been demonstrated for finger and hand movements as well as for elbow,^21^ arm (reaching and grasping,^22^^,^^26^^,^^27^^,^^28^^,^^29^ juggling,^30^ interceptive movements^31^), and whole body movements.^32^^,^^33^ Beyond touch, suppression of self-generated sensations is also evident in auditory,^34^ visual,^35^ and vestibular^36^ senses and across species.^37^^,^^38^

Tactile suppression is typically investigated by presenting near-^11^^,^^16^^,^^17^^,^^39^^,^^40^ or supra-threshold^13^^,^^41^ probe tactile stimuli on the limb that is about to be moved or moving. Tactile suppression has been shown during voluntary movement execution^1^^,^^2^^,^^42^ but also when the limb is passively moved,^2^^,^^43^ indicating a role for peripheral in addition to central mechanisms. Passive movements are aimed to mimic the kinematic characteristics of an active movement but are thought of lacking movement planning and generating associated motor commands that lead to sensorimotor predictions. As movement intention is absent or at least diminished during passive movements, efference copy signals are assumed to be substantially reduced.^44^ Consequently, the specific contribution of peripheral reafferent feedback on tactile suppression can be addressed. Previous studies typically found similar suppression for tactile stimuli presented right before an active or a passive movement,^16^^,^^21^^,^^22^ suggesting a role for peripheral mechanisms in tactile suppression. Peripheral mechanisms drive suppression by a postdictive process of backward masking, in which reafferent feedback from the movement masks weaker tactile stimuli that are typically used to probe tactile sensitivity on the moving limb.^21^ These findings stimulated an ongoing debate about whether movement-related tactile suppression stems from specific sensorimotor predictions,^39^^,^^40^^,^^45^ or an unspecific gating of tactile signals associated with voluntary movements.^46^^,^^47^

A drawback that comes with the comparison of the effects of active and passive movements on tactile suppression, though, is that passive movements were usually restricted (e.g., finger abduction in one defined direction^19^) and presented in blocks with fixed movement onset time so that participants could anticipate how and when their limb would be passively moved.^16^^,^^21^^,^^22^ This would allow one to predict the sensations associated with the limb that was about to be moved, similar to active movements, but without the involvement of motor commands arising from the movement. These expected sensations are *not* associated with internal forward models, but can result from non-motor predictions. Non-motor predictions refer to anticipation of an upcoming sensory input, irrespective of whether the input arises from an action. One such example is the ability to predict the timing of an upcoming stimulus on one’s own body, such as an external touch, when it is known which part of the skin is about to be stimulated, even when the stimulus is not associated with one’s own action. The exact identity of a stimulus, i.e., stimulus intensity can also be predicted even when it is not caused by an action.^48^ In addressing the role of central and peripheral mechanisms on tactile suppression, the impact of these non-motor predictions that are not related to internal forward models has so far been hardly considered.^48^^,^^49^ There is evidence that non-motor predictive processes result in attenuated perception of sensory stimuli, similar to central motor predictions generated by one’s own action.^50^^,^^51^ For example, sensory suppression can occur even in the absence of movement, whenever the sensory input can be anticipated, e.g., when a cue reliably precedes it.^52^^,^^53^ Other studies, however, found attenuated sensation for self-generated stimuli only for active movements, when the internal forward model could predict the upcoming sensory feedback, after taking into account non-motor predictive processes.^54^^,^^55^^,^^56^ Thus, by acknowledging the potential contribution of non-motor predictions on tactile suppression, we can gain a better understanding about the functional role of efference copy-related mechanisms.^55^^,^^56^^,^^57^^,^^58^^,^^59^

One way to test the impact of non-motor predictions is to manipulate the predictability that such sensations will occur: for example, when the occurrence of a sensory stimulus is predicted but no stimulus occurs or a stimulus of a different identity occurs.^40^^,^^60^^,^^61^ Such non-motor predictions can affect both passive and active movements. In passive movements, it can be argued that knowing how the movement will unfold and *feel like* might trigger predictions of sensory feedback associated with that limb’s movement and may lead to attenuated sensations on that limb. It is therefore likely that the predictability of an upcoming movement can engage non-motor predictions that could partially or substantially account for tactile suppression. Therefore, decreased tactile sensitivity prior to a passive movement may reflect the involvement of non-motor predictive processes associated with an upcoming movement, in addition to possible peripheral masking mechanisms. This possibility has not been considered in previous work.^16^^,^^21^^,^^22^

In the present study, we investigated the relative contributions of central motor predictive and peripheral masking mechanisms on tactile suppression while accounting for non-motor predictions common to both. To this end, we designed an experiment that involved active and passive movements of the right wrist. As tactile suppression can occur already shortly before the movement,^10^^,^^17^^,^^62^ we probed tactile sensitivity by presenting a brief vibrotactile stimulus of varying amplitude on the right hand shortly before movement onset. Participants made detection judgments under both movement conditions as well as under a baseline condition in which the wrist was resting. Based on existing evidence,^16^^,^^21^^,^^22^ we expected tactile suppression in active as well as in passive movements, as opposed to the baseline condition in which the limb was at rest. We also hypothesized that, if central motor predictive mechanisms play a role in tactile suppression,^39^^,^^40^ we would observe larger sensitivity decreases in the active compared to the passive movements. To explore the role of non-motor predictions in tactile suppression, we manipulated the predictability of a movement by presenting a cue in half of the trials, informing the participant about the upcoming condition (cued or cue+ baseline, active or passive) before each trial. In a separate block, these conditions were presented without an instruction (uncued or cue− baseline, active or passive). For active movements, in which the associated sensations are predictable even when not cued (see STAR Methods), the cue effect should be negligible or nonexistent, while for passive movements, it should be present. By manipulating the predictability of an upcoming passive movement, we were able to address the potential role of non-motor predictions on tactile suppression. If non-motor predictions boost tactile suppression, suppression should be larger when the upcoming passive movement (and its associated sensory feedback) was 100% predictable, i.e., when *cued* before execution compared to when the movement was partially (50%) predictable. Assuming that cued passive movements reliably involve non-motor predictions about the upcoming sensory feedback associated with the movement, we expect to observe stronger suppression in the passive cued condition (fully predictable) than in the passive uncued condition (partially predictable) in comparison to the active cued vs. uncued conditions.

## Results

### Signal detection measures

**Figure 1:**
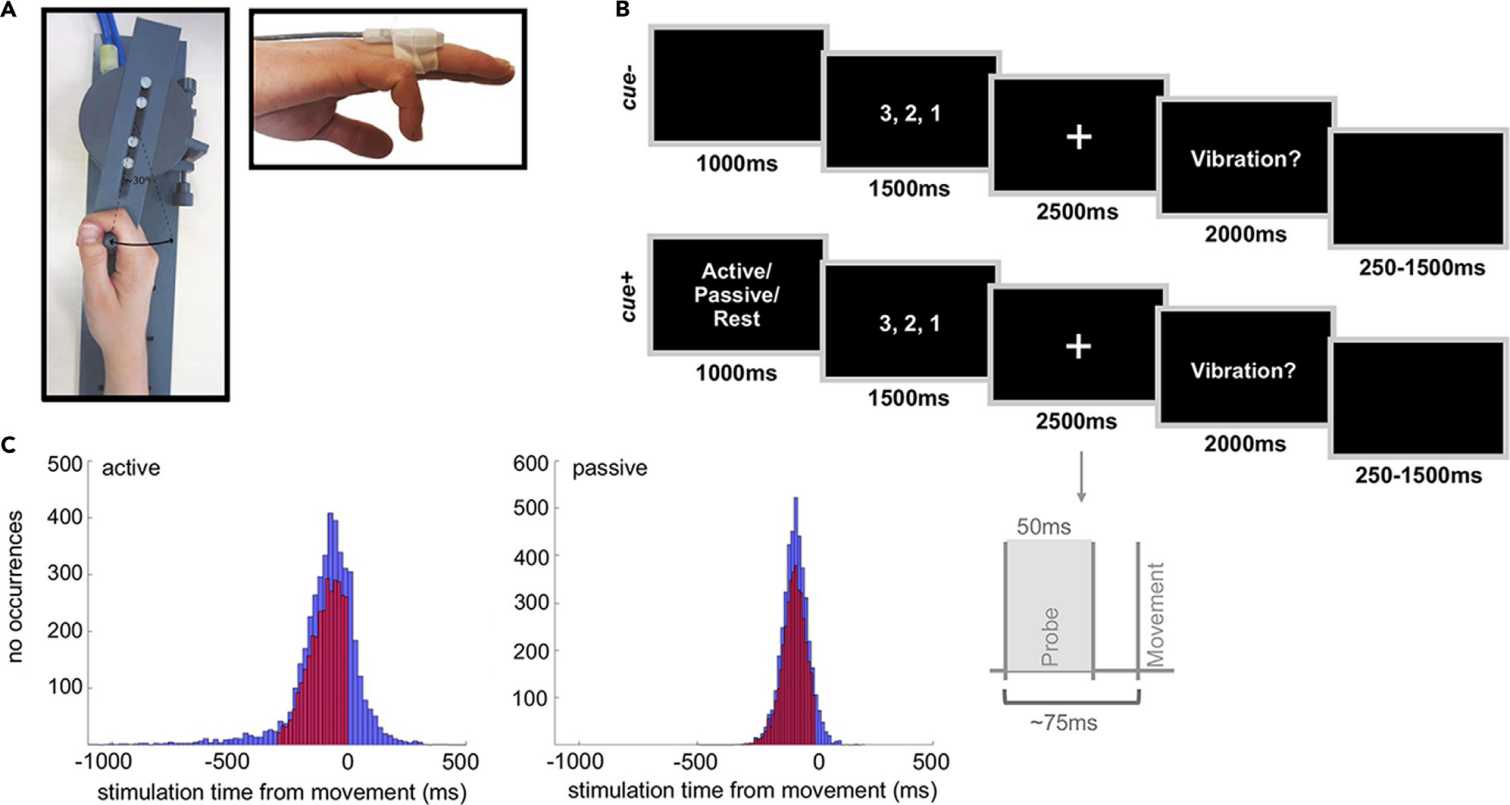
Experimental apparatus and procedure (A) Experimental setup. Custom-made PMD. Movement trajectory was along the horizontal arc, and the range of movement from one end to another was 30°. Tactile stimulus was presented by the piezotactor attached to the dorsal part of the right ring finger. (B) Timeline of an experimental trial in the cue− and cue+ blocks. In cue− blocks, participants were instructed to either rest or perform a wrist movement or let the device perform the movement after a countdown period. They could receive a tactile stimulus of variable intensities right before the active/passive movement or during rest. They were then asked to respond whether they detected the stimulus or not. Cue+ blocks were exactly the same except that the participants received an instruction on the movement type (active, passive or rest) at the beginning of each trial. (C) Histograms depicting the distribution of stimulation time relative to movement onset for active and passive trials. In all analyses regarding tactile suppression, we included only trials with tactile stimuli presented from 300ms before movement onset until the moment of movement initiation (highlighted in red).

**Figure 2:**
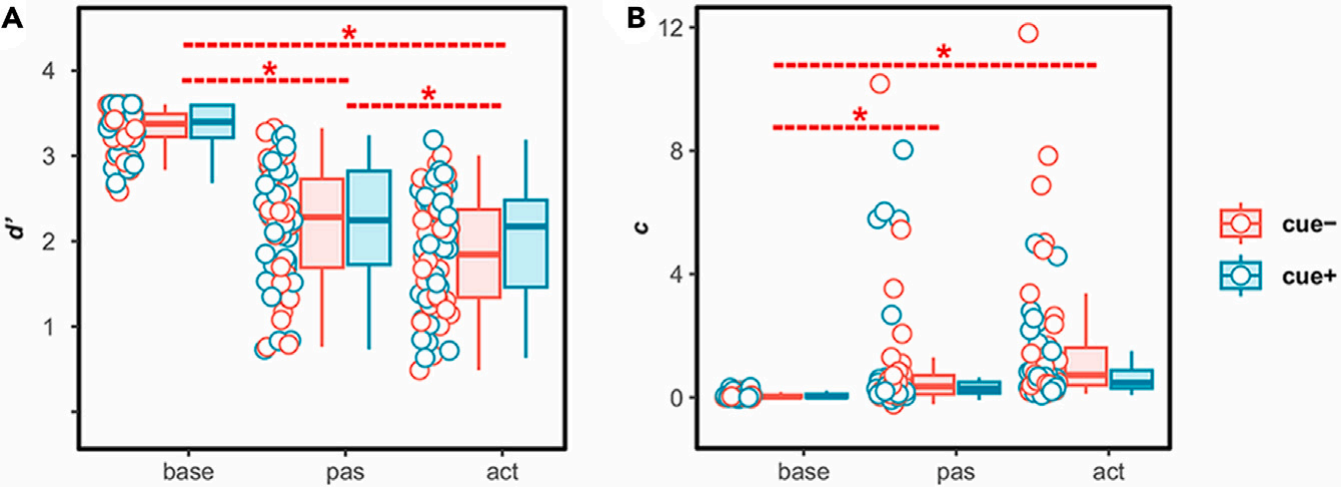
Plots depicting tactile sensitivity and response criterion (A) Tactile sensitivity (*d'*) and (B) Response criterion (*c*) as a function of movement and cue. Higher *d'* values indicate increased sensitivity to tactile stimulus whereas higher *c* values indicate increased tendency to respond to stimulus as absent. Dots show individual data points collapsed across binned amplitudes whereas boxplots depict group-level means with error bars showing standard deviations; solid lines show median of the points (*n* = 35 in cue- and *n* = 33 in cue+). Significant differences revealed by the LMMs (*d'*: *F*(2, 928.45) = 473.24, *p* < 0.001; *c*: *F*(2, 929.65) = 21.91, *p* < 0.001) are indicated by bold asterisks.

Participants were instructed to detect a brief vibrotactile stimulus on the right ring finger while resting (baseline), shortly before moving their right wrist (active) via a passive movement device (PMD) handle or shortly before their right wrist was moved by the PMD (passive) (see Figure 1, see also Figure S6 for the distribution of stimulation onsets relative to movement onsets in each condition). They did so in two separate blocks in which the upcoming condition (i.e., passive or baseline) was instructed (cued, cue+) or not (uncued, cue−). To test the role of movement (baseline, active, passive) and cue (cue−, cue+) on tactile suppression, we computed a linear mixed model (LMM) on tactile sensitivity, as reflected in *d',* with movement, cue and their interaction as fixed effects of interest, stimulus amplitude as covariate and participant as random effect. The results can be found in Table 1. The LMM showed a main effect of movement. Post-hoc pairwise comparisons revealed that tactile sensitivity was significantly lower in the active (lower CI = 1.76, upper CI = 2.09) (estimate = 1.41, *t* = 28.89, *d* = 1.90, *p* < 0.001), and the passive conditions (lower CI = 2.03, upper CI = 2.35) (estimate = 1.15, *t* = 23.39, *d* = 1.54, *p* < 0.001) as opposed to the baseline condition (lower CI = 3.18, upper CI = 3.50), demonstrating the well-established tactile suppression effect. Importantly, tactile sensitivity was significantly lower in the active compared to the passive condition (estimate = −0.26, *t* = −5.33, *d* = −0.35, *p* < 0.001), revealing a stronger suppression when movements were voluntarily initiated. There was no main effect of cue, nor was there a significant interaction between cue and movement (see Figure 2A). Finally, a significant main effect of stimulus amplitude was found (see Table 1).

**Table 1:** Fixed effects of interest from the LMM on *d'*

Sensitivity (*d'*)	*SS*	*MS*	*dfs*	*F*	*p*
Movement	369.16	184.58	2, 928.45	473.24	<0.001
Cue	0.93	0.93	1, 943.71	2.39	0.12
Movement x cue	0.68	0.339	2, 928.40	0.87	0.42
Amplitude (covariate)	190.48	190.48	1, 928.52	488.36	<0.001

Statistics are reported for each item with sum of squares (*SS*), mean squares (*MS*), degrees of freedom (*dfs*), F-value (*F*) and *p* value (*p*).

We conducted a similar LMM for the response criterion as reflected by *c*, with movement, cue and their interaction as fixed effects of interest, stimulus amplitude as covariate and participant as random effect. The results can be seen in Table 2. There was a significant main effect of movement. Post-hoc pairwise comparisons revealed significantly smaller *c* values for the baseline (lower CI = −0.35, upper CI = 0.43) as opposed to the active (lower CI = 0.99, upper CI = 1.77) (estimate = −1.34, *t* = −6.49, *d* = −0.43, *p* < 0.001) and the passive condition (lower CI = 0.54, upper CI = 1.33) (estimate = −0.90, *t* = −4.32, *d* = −0.28, *p* = 0.001). There was no significant difference between active and passive conditions, nor was there a significant interaction between cue and movement (see Figure 2B). Finally, a significant main effect of stimulus amplitude was found (see Table 2).

**Table 2:** Fixed effects of interest from the LMM on *c*

Criterion (*c*)	*SS*	*MS*	*dfs*	*F*	*p*
Movement	306.47	153.24	2, 929.65	21.91	<0.001
Cue	12.16	12.16	1, 961.27	1.74	0.19
Movement x cue	34.81	17.41	2, 929.44	2.49	0.08
Amplitude (covariate)	497.55	497.55	1, 930.02	71.14	<0.001

Statistics are reported for each item with sum of squares (*SS*), mean squares (*MS*), degrees of freedom (*dfs*), F-value (*F*) and *p* value (*p*).

### EMG data

**Figure 3:**
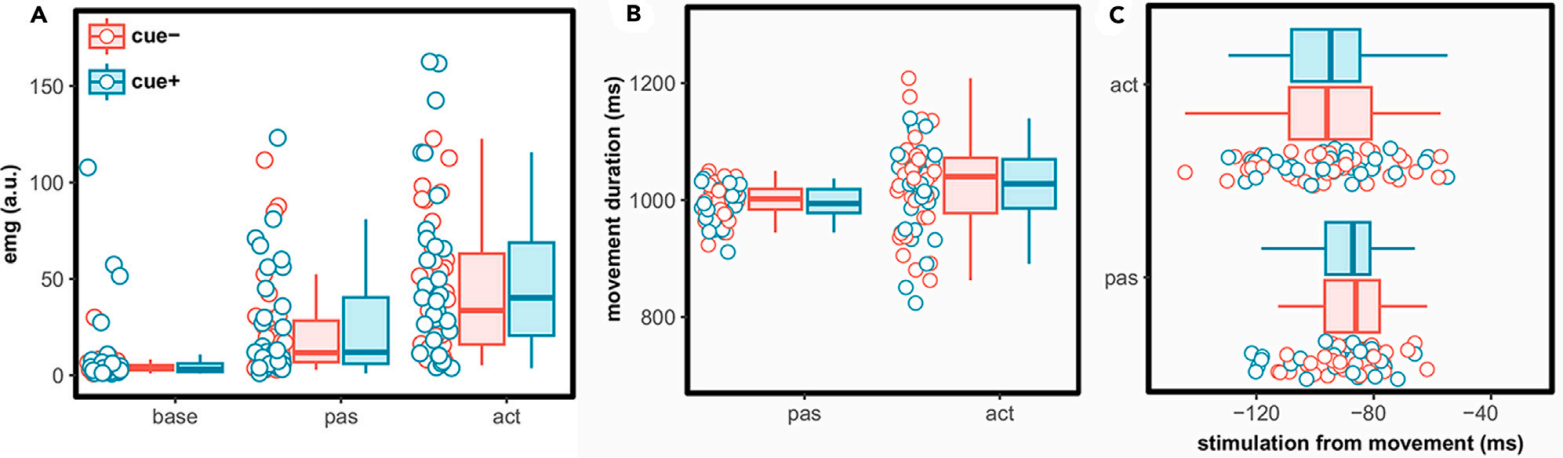
Plots depicting muscle activity and kinematic measures (A) EMG activity as a function of movement and cue (a.u. arbitrary units; *n* = 34 in cue− and *n* = 30 in cue+). (B) movement duration (in ms; *n* = 35 in cue- and *n* = 33 in cue+) and C. stimulation time from movement onset (in ms; *n* = 35 in cue- and *n* = 33 in cue+) as a function of movement and cue. Negative values indicate stimulation occurring earlier than movement. Dots show individual data points collapsed across binned amplitudes whereas boxplots depict group-level means with error bars showing standard deviations; solid lines show median of the points.

We calculated EMG activity in each condition by subtracting the maximum EMG amplitude (of 10 maximum values) in each condition from the pre-stimulus baseline activity (1000ms). Mean difference values for each condition were subjected to an LMM with movement, cue and their interaction as fixed effects of interest and participant as random effect. The type of movement had a significant effect with post-hoc pairwise comparisons revealing significantly larger EMG values for the active (lower CI = 40.70, upper CI = 57.09) as opposed to the baseline (lower CI = −0.35, upper CI = 15.96) (estimate = 41.1, *t* = 8.61, *d* = 1.4, *p* < 0.001) and the passive condition (lower CI = 16.24, upper CI = 32.55) (estimate = 24.5, *t* = 5.14, *d* = 0.84, *p* < 0.001). The EMG signal during passive movements was on average two times smaller during active movements. There was also a significant difference between passive and baseline conditions (estimate = 16.6, *t* = 3.49, *d* = 0.57, *p* = 0.002). There was no significant difference between cue conditions or interaction (see Figure 3A; Table 3).

**Table 3:** Fixed effects of interest from the LMM on *EMG*

EMG (a.u.)	*SS*	*MS*	*dfs*	*F*	*p*
Movement	53290	26645	2, 145.89	37.52	<0.001
Cue	2481	2481	1, 160.74	3.49	0.06
Movement x cue	72	36	2, 145.89	0.05	0.95

Statistics are reported for each item with sum of squares (*SS*), mean squares (MS), degrees of freedom (*dfs*), F-value (*F*) and *p* value (*p*), a.u. arbitrary units.

### Kinematic data

To examine differences in stimulus presentation and kinematic parameters across conditions, we conducted two separate LMMs on these variables, looking for potential differences based on movement type (active vs. passive), cue (cue−, cue+) and their interaction. We focused on the stimulation onset relative to movement onset and movement duration (see Figures 3B and 3C). The results can be found in the supplemental information (Tables S1 and S2). First, we found significant differences between active and passive movements in terms of when the vibrotactile probe stimulus was presented relative to movement onset. On average, the vibrotactile probe stimulus occurred just 6ms earlier in the active (lower CI = −100.47, upper CI = −91.69, mean = −96.06) than in the passive (lower CI = −94.84, upper CI = −86.02, mean = −89.95) (estimate = −5.65, *t* = −2.81, *d* = −0.23, *p* = 0.005) condition, always relative to the movement onset. Second, movement duration was on average 28ms longer for active (lower CI = 1008.07, upper CI = 1038.42, mean = 1024.68) than passive (lower CI = 979.35, upper CI = 1009.76, mean = 996.40) (estimate = 28.7, *t* = 7.30, *d* = 0.59, *p* < 0.001) movements. Given that passive movements were much less variable in terms of kinematic parameters, as they were initiated by a device and thus motor noise was substantially eliminated, such differences between active and passive movements were expected. Nevertheless, the question remains whether these small but systematic differences in these parameters could have contributed to the differences in tactile sensitivity or response criterion between conditions. To address this, we examined the potential relationship between tactile sensitivity (*d'*) and response criterion (*c*) with stimulation onset and movement duration by calculating eight separate Spearman correlation coefficients per parameter. The results are listed in the supplemental information (Figures S4 and S5). Overall, we did not find any significant correlations between these measures. Thus, differences in stimulation onset and movement duration across active and passive movements as well across cue conditions are unlikely to influence tactile processing and therefore signal detection measures.

## Discussion

In the present study, we investigated the role of central motor and peripheral mechanisms on tactile suppression while taking potential non-motor predictive processes into account. We compared tactile perception during active and passive movements which allowed us to address the relative contributions of efferent signals associated with sensorimotor predictions and movement-related reafferences that may hinder the detection of vibrotactile stimuli through masking.^16^^,^^21^^,^^22^ To this end, we probed tactile perception just before movement onset, at a moment when active movements are supposed to involve motor predictions that lead to predictive tactile suppression.^24^^,^^25^^,^^28^^,^^39^ Our results show that tactile suppression is evident in both active and passive movements, but importantly suppression is greater during active than passive movements. Together, we demonstrate that tactile suppression is mediated by distinct central predictive mechanisms, further supporting recent findings,^39^^,^^40^ and clarifying previous contradictory results^16^^,^^21^ and assumptions.^46^^,^^47^

Using bias-free measures of perceptual sensitivity,^63^^,^^64^ we found decreased sensitivity to vibrotactile probes during both active and passive wrist movements compared to when the wrist was resting, in line with previous findings showing that movement leads to suppression.^4^^,^^5^^,^^26^ Importantly, and in contrast with what previous studies have reported,^16^^,^^21^^,^^22^ suppression was significantly larger for active compared to passive movements. Previous work showed similar tactile suppression for active and passive movements, suggesting an important role for peripheral mechanisms that can account for tactile suppression.^16^^,^^21^^,^^22^ Two of these studies also looked into the time course of suppression, as its early onset underlines the role of central motor predictions in downregulating movement-related tactile input.^16^^,^^25^^,^^62^ The time course of suppression seems to show a similar pattern across active and passive movements, though this has been only shown for stimulation on the finger before its movement^16^^,^^21^ whereas when the moving effector is distal to the stimulation site (elbow movements coupled with finger stimulation), suppression has been found to begin earlier for active as opposed to passive movements.^21^ In our study, the moving effector was close to the digit receiving the tactile probe. Contrary to previous findings, we observed a clear difference in tactile suppression between active and passive movements, even after taking the potential role of non-motor predictive processes into account, such as predictability of the quality and timing of sensations associated with passive wrist movement.^48^ In order to test if the time course of tactile suppression differed across active and passive movements, we conducted an additional analysis on the detection rates of stimulation trials binned in terms of time of stimulation relative to movement onset. The results showed earlier decline in the detection of vibrotactile stimuli for active compared to passive movements (see supplemental information for details and Figures S6 and S7; Table S3). These findings indicate a distinct role for central motor predictions in downregulating tactile sensitivity around human movement. Peripheral mechanisms may still play some role, as suggested by the presence of tactile suppression in passive movements. However, it cannot solely account for suppression observed shortly before or during active movements. Together, our results highlight the role of central motor predictions in tactile suppression, questioning previous findings of similar suppression effects in active and passive movements and challenging recent accounts that postulate tactile suppression independent of motor predictive processes.^46^^,^^47^

Decreased sensitivity during active and passive movements was accompanied by a tendency to report absence of vibrotactile probes when there was a probe present (miss trial), confirming a shift in response criterion in the presence of a movement compared to resting. This is in line with previous work^30^^,^^65^^,^^66^ showing an increased tendency to report the absence of a tactile stimulus when those stimuli are present. The shift in response criterion was similar across active and passive movements. Therefore, differences in sensitivity across active and passive movements are unlikely to result from a change in response criterion, as *c* values were comparable. It can be speculated that response criterion shifts observed during tactile suppression were mediated by peripheral rather than central motor-related mechanisms, as peripheral signals were present across both movements. Finally, the present design cannot address the exact nature of response criterion differences,^67^ namely, if they are perceptual or decisional.

Tactile suppression can be modulated by the temporal proximity of the probe stimulus to the movement^17^^,^^26^ and by the movement speed.^68^ (but see also the study by Fraser et al.^69^) We presented the tactile probe stimulus right before movement onset, i.e., in the time window in which movement-related reafferences were absent and efferent signals present. Our experimental setup allowed us to determine the time of stimulus occurrence relative to movement onset that was slightly earlier for active compared to passive movements (difference of 6ms). In addition to temporal proximity of stimulus to the movement, differences in movement speed between active and passive movements can potentially explain differences in tactile suppression.^68^ Although our setup did not allow us to estimate movement speed, we calculated the total movement duration as a proxy of movement speed. Overall, active movements resulted in systematically longer movement duration than passive movements. As passive movements were guided by the device and thus were less variable than active movements in terms of movement onset and duration, these findings were expected. If stimulus onset and movement duration were to influence our results, these would be expected in the opposite direction. For example, one would expect larger suppression for passive movements as stimulus onsets were closer to movement onset.^16^^,^^21^ Moreover, if considered as a proxy to movement speed, shorter movement durations in the passive condition would likely come with higher movement speeds and this should have led to stronger tactile suppression than in the active condition.^68^^,^^70^ In order to address the potential influence of these parameters on tactile suppression, we conducted correlation analyses between stimulus onset, movement duration, and signal detection measures. No significant correlations between these measures were found (all *p* > 0.05), suggesting that these are unlikely to explain differences in tactile suppression across movements.

We further aimed to determine the effect of non-motor predictive processes on tactile suppression because predictions not specific to the internal forward model can also result in the downregulation of sensitivity.^54^^,^^55^^,^^56^^,^^57^^,^^58^ We hypothesized that tactile suppression should be influenced by non-motor predictions as a result of predictability of sensory feedback associated with an upcoming movement. We tested this hypothesis in passive movements by cuing or not cuing the participants about whether the upcoming trial involved a passive movement. In the cued block, the passive movement and therefore non-motor aspects of sensory consequences of the movement, i.e., imminent sensations^48^^,^^49^ were fully predictable (100%).^55^ In contrast, in the uncued block, passive movements were partially predictable (50%), so the role of non-motor predictions could be addressed by comparing potential differences in tactile suppression as a function of predictability. If non-motor predictions play a role in the resulting sensation, tactile suppression in the cued passive condition should be greater than in the uncued passive condition. We expected such a cue effect in the passive movements but not in the active movements. We did not find a difference between cued and uncued passive movements. Our results expand previous work showing similar attenuation profiles for self-generated visual and auditory stimuli when controlling for non-motor predictions.^54^^,^^55^^,^^56^^,^^57^^,^^58^

Identifying the source of sensations is essential to respond efficiently to changes in our environment such as unexpected events, potential obstacles, or threats. Through suppression of predicted tactile sensations caused by self-generated movements, the nervous system can modulate bodily sensations associated with the movement, differentiate self-produced from externally-produced stimuli and efficiently respond to unexpected, novel stimuli despite its limited capacity. Tactile suppression is a well-established phenomenon observed across sensory modalities and species.^34^^,^^35^^,^^36^^,^^37^^,^^38^ For instance, humans attenuate self-vocalizations compared to external vocalizations.^54^^,^^71^^,^^72^ In mice, perception of auditory feedback arising as a result of self-locomotion is suppressed.^38^ This systematic decrease in sensitivity has been explained by central efference copy mechanisms predicting upcoming sensorimotor states associated with voluntary movements.^37^^,^^49^ However, non-motor predictions that may modulate perceptual sensitivity associated with voluntary movements have only recently been explored.^48^^,^^49^ By comparing active and passive movements in a paradigm that addresses the potential contribution of non-motor predictions, we demonstrate the role of efference copy signals in tactile suppression. Our results support the notion of such a central predictive mechanism in the domain of touch that downregulates anticipated sensations associated with voluntary movements. The present findings contribute to our understanding of sensorimotor control and predictive mechanisms and their dysfunction in clinical populations such as patients with Parkinson’s disease^73^ and schizophrenia.^74^^,^^75^ In addition, elucidating the interplay between central predictive and peripheral signals on human sensorimotor performance is relevant to address the potential benefits of these movements in other movement-related disorders.^76^

### Limitations of the study

This study has some limitations. The first limitation concerns the potential involvement of motor command signals in passive movements. During passive movements, participants had to maintain a grip on the PMD handle that may have led to small, corrective movements of the fingers and the wrist. This raises the possibility that some level of muscular involvement and motor command signals were present during passive movements. To reduce muscular involvement, we carefully instructed participants to maintain a relaxed hand posture throughout the experiment and monitored the ease of the PMD handle in executing passive movements given the assumption that corrective movements potentially lead to the PMD handle not moving in the expected direction. We also compared EMG activity on the right extensor carpi radialis longus during active and passive movements. EMG activity was substantially reduced during passive movements compared to active movements, indicating a substantially higher level of motor involvement during active than passive movements. We can conclude that our setup allowed us to reduce motor involvement during passive movements, although some level of motor involvement may be present. The second limitation concerns the level of predictability of the passive movement that we could have induced. This may have resulted in the cue effect being weaker than it may actually be. In our experimental paradigm, the least predictable condition was at chance level (50%), with the expectation that it was equally strong for an upcoming passive movement and a baseline trial in which the limb was at rest. It can be argued that the effect of non-motor predictions on tactile suppression can be detected with lower predictability levels (less than 50%). In future studies, non-motor predictions can be scrutinized by reducing predictability to less than chance levels, for example, by decreasing the ratio and therefore predictability of a passive movement by introducing a no-movement condition in which the movement is expected but not taken place^25^ or by manipulating the timing or identity of the sensory feedback associated with the movement.^48^^,^^61^ The third limitation concerns the potential influence of attention on tactile suppression. It is possible that movement preparation can deploy attentional resources to the to-be-moved location, modulating perceptual sensitivity.^41^^,^^77^^,^^78^ In our paradigm, the stimulation was on the moving effector, delivered shortly before movement initiation. Based on existing evidence showing facilitation of perception by spatial attention,^77^^,^^78^ one would expect increased tactile sensitivity to stimuli delivered on the same limb in active as opposed to passive movements. We observed the opposite pattern in which active movements resulted in reduced tactile sensitivity compared to passive movements. This is in line with existing evidence showing robust tactile suppression under attentional constraints.^41^^,^^79^ Second, attention may exert its effect as a reduction in tactile sensitivity due to additional task demands. In the active condition, participants had to plan the movement and detect the tactile stimulus whereas in the passive condition, they mainly had to detect the stimulus. Increased task demands could lead to reduced tactile sensitivity in the active compared to the passive condition. Although our results could be interpreted as such, existing evidence shows robust tactile suppression under increasing task demands.^8^^,^^41^^,^^79^^,^^80^ Although attention seems to be unlikely to account for the present results, more evidence is needed to confirm whether active and passive movements lead to different attentional constraints that may influence perceptual sensitivity.

## STAR★Methods

### Key resources table

**Table:** 

REAGENT or RESOURCE	SOURCE	IDENTIFIER
**Deposited data**		

Behavioral data	This study	www.osf.io/3qkxj/

**Software and algorithms**		

Brain Vision Recorder and Analyzer	Brain Products GmbH, Munich, Germany	www.brainproducts.com
GPower Release 3.1.9.7	Faul et al.^81^^,^^82^	www.psychologie.hhu.de
Microsoft Visual C++	Visual Studio Windows	www.microsoft.com
Matlab 2019a	The MathWorks Inc.	www.mathworks.com
R Studio 2023 4.2.3	RStudio Team	www.rstudio.com
Ogre Framework version 1.7.4	The MIT License (Torus Knot Software Ltd.)	www.ogre3d.org

**Other**		

Pneumatic movement device	Custom	N/A
Tactors	Engineering Acoustics	www.eaiinfo.com
BrainAmp ExG MR (amplifier)	Brain Products GmbH, Munich, Germany	www.brainproducts.com

### Resource availability

#### Lead contact

Further information and requests for resources should be directed to and will be fulfilled by the lead contact, Belkis Ezgi Arikan arikan.ezgi@gmail.com.

#### Materials availability

Detailed description of the materials is listed in the STAR Methods file. Any further information is available from the lead contact upon request.

#### Data and code availability

• Data: De-identified human behavioral data have been deposited at Open Science Framework and are publicly available as of the date of publication. Accession link is listed in the key resources table.
• Code: This paper does not report original code.
• Additional information: Any additional information required to reanalyze the data reported in this paper is available from the lead contact upon request.

### Experimental model and study participant details

We recruited 37 naïve participants (28 females, mean = 24±4 years old). We aimed for a minimum sample size of N = 34 based on an a-priori power analysis using G∗Power3.1^81^^,^^82^ to test the difference between two dependent group means using a two-tailed t-test for a medium effect size of interest (*d* = 0.5) with an alpha of 0.05 to achieve a power of 0.80. The participants were right-handed as confirmed by the Edinburgh Handedness Inventory.^83^ They reported no neurological and/or psychiatric conditions and had normal or corrected-to-normal vision. The experiment was approved by the local ethics committee of the University of Marburg (Ethikkomission, Fachbereich Medizin, Studie 157/21) and was performed in accordance with the Declaration of Helsinki except for pre-registration.^84^ All participants gave written informed consent prior to participation.

### Method details

#### Environment and apparatus

The experiment took place under normal lighting conditions. Participants sat in front of a computer screen (1920 x 1200pixel resolution, 60Hz frame rate, 24″ screen size) with ∼50cm viewing distance. The participant’s right hand was placed on a custom-made passive movement device (PMD) which was used to perform active and passive movements. The handle of the PMD was used for the movement execution. The movement had a horizontal trajectory with a constant range of ∼30° and ∼5.5cm (see Figure 3A). Motion to the PMD handle could be induced automatically with compressed air (3 bar). Approximate force that had to be used to move the handle automatically was 30N. An optical fiber attached to the PMD tracked the position of the handle in real time. The timing of experimental events including movement information from the PMD were implemented and recorded by a custom-made software written in C++.

Stimulus detection responses were recorded via a keyboard (‘V’ and ‘N’ buttons on the keyboard). There were 132 trials for each condition. On half of all the trials, vibrotactile stimuli of 11 different amplitudes (240Hz frequency, 50ms duration) were presented on the dorsal side of the right ring finger using a piezotactor (Engineering Acoustics, Casselberry, FL). The stimulus amplitude was defined as the peak-to-peak displacement of the tactor and ranged from 0.013 to 0.140mm in steps of 0.013mm, each repeated six times per condition (movement and cue). The amplitude range was based on a series of pilot studies in which we aimed to capture near-threshold stimulus range for the stimulus characteristics and the movement type used in the experiment prior to the main experiment (see Figure S1; Figure S2 for individual detection performance; Figure S3 for detection thresholds across participants). The remaining half of the trials did not involve any stimulus.

A uniform black background was used as a screen background, and the instructions were presented in white color (except for the countdown in the active cue + condition; see below). The right hand of the participant was occluded by a partition to prevent any visual feedback about the wrist movement that could influence the performance in the detection task. In addition, to prevent participants from using auditory cues that might arise from the PMD or the tactor, participants wore earplugs, and were additionally presented with white noise via headphones throughout the experiment.

#### General procedure

Participants completed the experiment on two separate days (sessions). In one session, they were instructed in the beginning of each trial on the type of movement (baseline, active or passive; = cue+ condition), whereas in the other session, they received no cue (baseline, active or passive; = cue- condition). Session order was counterbalanced across participants.

Each session consisted of two blocks, in which participants were presented with baseline, active and passive conditions in randomized order. In each block, they were asked to hold the handle of the PMD using their thumb, index and middle fingers while relaxing their wrist and arm. In the baseline condition, participants were instructed to rest their right wrist in the instructed position. In the active condition, they were asked to move their right wrist via the PMD from the very left to the very right and then back to the start position. In the passive condition, the PMD moved the participant’s wrist in the same trajectory as in the active condition. In each trial of all conditions, participants could receive a tactile stimulus varying in amplitude or no stimulus, to which they reported via the keyboard whether they detected it or not. This tactile detection task happened ∼1000 ms at the end of the movement or ∼1800ms after the stimulus presentation in the baseline condition.

Timeline of an experimental trial is depicted in Figure 1B. Each trial began with a 1000ms period which was either used to instruct the participant of the upcoming movement (cue+ condition; baseline, active or passive) or could be blank (cue- condition; baseline, active or passive). Immediately after that, a countdown period began in which the numbers 3-2-1 were presented subsequently on the screen, each for 500ms. Participants were instructed to use this period to be prepared to move if the condition was active. In order for the participant to plan the movement in the cue- condition, we presented the numbers in gray when the trial was active. For passive trials, participants could form predictions on the upcoming passive movement in the cue+ condition whereas this was less likely for the cue- condition as they could expect either a passive movement or no movement (baseline). The countdown was followed by a white fixation cross that remained on the screen for 2500ms. In the baseline condition, participants remained still during this period, and in half of the trials they were presented with a vibrotactile probe stimulus 698 ± 90ms (mean ± SD across participants) after the countdown. In the active condition, participants performed a wrist movement for a duration of ∼1000ms anytime within this period. We encouraged the participants to perform the movement not as an automatic response to the occurrence of the fixation cross,^85^ but to initiate the movement at a time of their choice, provided that they maintained more or less the same movement onset time across trials. This restriction was necessary for the stimulus presentation to be comparable within and across movements, since stimulus presentation in the active and passive movements were based on active movement onsets (see below). In the active condition, the vibrotactile probe stimulus was presented on average 95 ± 139ms (mean ± SD across participants) prior to the movement onset (see Figure 1C). In the passive condition, the PMD moved the participant’s wrist in the same trajectory as in the active trials. The vibrotactile probe stimuli in passive trials were presented 86 ± 54ms (mean ± SD across participants) before the movement onset (see Figure 1C). After the countdown and the fixation period, the question ‘Vibration?’ appeared on the screen. The participants used the keyboard to register their ‘yes’ or ‘no’ responses and had 2000ms to do so. The trial ended with an inter-trial interval of 750ms (range: 0-1500ms). EMG was recorded from the right extensor carpi radialis longus muscle (see EMG acquisition and preprocessing).

In all movement trials involving vibrotactile probes, we aimed to present the vibrotactile stimulus ∼75ms prior to the movement onset when tactile suppression should be substantial and movement-related reafferences minimal.^10^ For this, we initially presented participants with 12 practice trials in which they had to perform active movements. The movements were initiated after the countdown period and should not exceed the time window of 2500ms that was allotted for the movement execution. The vibrotactile stimuli in the first five practice trials were presented 75ms after the countdown, regardless of the participant’s movement onset. We used these first five trials to compute a mean movement onset estimate for the following trial.^28^^,^^29^ Stimulation onsets in the subsequent practice trials were then adjusted according to the movement onsets from previous five trials. These practice trials were excluded from all analyses. This procedure provided robust estimates of movement onset, as probes were presented before movement onset in 75% of all active trials that were completed as instructed, and in 94% of all passive trials that were completed as instructed. Moreover, in order to maintain comparability across movements while accounting for variability within trials, we extracted the mean and standard deviation of movement onset times from the active trials and randomly draw a stimulus onset time from this distribution to present the vibrotactile stimuli in the passive condition. The participants were trained prior to the experiment on how to perform the active movement within 1000ms. For this, we used a metronome and asked the participants to move the PMD handle according to the metronome’s pace. They also received training on the experimental block and were given feedback on their performance. There were two identical blocks within a session and the participants were encouraged to take a break in between. The entire procedure took ∼2 h/session.

#### EMG acquisition and preprocessing

EMG data were recorded with Brain Vision Recorder and compatible hardware (Brain Products GmbH, Munich, Germany). Bipolar surface electrodes were placed longitudinally over the right extensor carpi radialis longus with an inter-electrode distance of ∼2cm. The ground electrode was positioned on the right wrist joint. The electrodes were placed after treating the area with alcohol abralyte gel (EasyCap, Munich, Germany) and removing the hair when necessary. The electrode cables were fixed with medical tapes to prevent interference artifacts and were attached to an electrode input box, which was connected to an amplifier (BrainAmp ExG MR). An acceleration sensor was fixed on the right corner of the PMD to detect movement of the PMD handle. EMG signals were sampled at 1000Hz, filtered (bandpass: 10±1000Hz), amplified (input impedance = 20kΩ) and digitized with a 16bit A/D converter. The data were stored on a personal computer for offline analysis.

EMG activity for each movement trial was calculated from the filtered and smoothed signal. Movement onset and offset were determined by the accelerometer (y-axis). For each movement trial, we took the maximum amplitude of the EMG signal (mean of the largest 10 values) starting from 500ms before and 500ms after movement onset and offset, respectively. These amplitudes were baseline corrected by a 1000ms pre-stimulus phase extracted from the instruction phase of the corresponding trial. For each participant, we took the baseline-corrected values to calculate three mean difference signals, for baseline trials as well as active and passive movements and conducted the group-level statistical analysis on the mean difference values.

#### Signal detection analysis

Signal detection framework explains how an observer detects the presence or absence of a sensory stimulus. It conceptualizes stimulus detection as a combination of two processes in which perceptual evidence is gathered (sensitivity) and a decision is made (response criterion). Sensitivity refers to the internal representation of a stimulus given noise, whereas response criterion refers to the tendency to respond in a certain way, e.g., a bias to respond ‘no stimulus detected’.^86^ Signal detection framework allows one to address the relative contributions of these processes. We used Matlab 2019a (The MathWorks Inc., Natick, MA) to quantify individual signal detection measures of sensitivity (here *d'*) and *c* scaled to *d'* (here relative *c*) in cases where *d'* varies across conditions.^30^^,^^86^ Accordingly, for each movement and cue condition, we extracted all valid signal (trials with vibrotactile stimulus) and noise (trials without vibrotactile stimulus) trials. Because tactile suppression has been found to take place up to 300ms before movement onset,^11^^,^^29^^,^^87^ we included only those signal trials in which the maximum time between stimulation and movement onset was 300ms. For each movement and cue condition, we grouped the stimulus amplitudes into five bins (12, 12, 16, 12, 12 trials per bin), starting from the lowest amplitude that was presented (average amplitudes per bin: 0.019, 0.044, 0.076, 0.107, 0.133mm). Each bin involved two subsequent amplitudes, except for the third bin that involved three subsequent amplitudes. For simplicity, we will refer to this variable as *amplitude*. The stimulus amplitude range was chosen to be both within and above a detection threshold (point in which the participant detected the stimulus 50% of the time, see also Figures S1 and S2 for individual detection threshold estimates in each condition). We included a slightly larger amplitude range for medium amplitudes as there may be inter-individual differences around the detection threshold. Similar to the signal trials, we divided the noise trials into five bins. For each amplitude, movement and cue condition, we used the signal and noise trials to calculate hit rate (‘yes’ responses in trials when signal is present/all signal present trials) and false alarm rate (‘yes’ responses in trials when signal is absent/all signal absent trials). We then transformed the hit and false alarm rates into z-scores to calculate *d'* and *c* for each binned amplitude.^64^ In the case of perfect accuracy (hit rate of 1) or no false alarms (false alarm rate of 0) which would result in the z-transform score to reach infinity, we implemented the loglinear approach to adjust the hit and false alarm rates.^88^ Accordingly, we added 0.5 to both the number of hits and the number of false alarms, and added 1 to both the number of signal trials and the number of noise trials, respectively.

#### Analysis on kinematic data

Kinematic data was analyzed with Matlab 2019a. For each movement trial, we calculated movement onset and movement duration. Movement onset was determined based on the relative position of the PMD handle from the start position. Accordingly, movement onset was defined as the time point in which the position of the handle exceeded 2 units of the quadrature encoder inside the PMD. Similarly, movement offset was defined as the time point in which the position of the PMD handle was positioned on the left side up to 2 units away from the start position. Movement duration was defined as the total time (in ms) between movement onset and offset.

#### Rejection of trials and conditions

We employed a two-step exclusion criterion to the data. First exclusion criterion concerned the participant’s behavior and the setup. We excluded trials from further analyses if the participant failed to follow the instructions (e.g., moving in a baseline trial) and in the case of a technical problem with the setup (e.g., movement algorithm did not work). Accordingly, data from one participant’s session was discarded due to failure to follow the instructions. We also discarded responses from those trials in which the participant failed to perform the instructed movement or did not respond to the question regarding vibration (5% of all trials). Second exclusion criterion concerned task performance that could allow us to draw reliable conclusions from the data. For this, we discarded the trials in which suppression is unlikely to take place,^10^^,^^16^^,^^26^^,^^62^ i.e., trials in which the probe was presented earlier than 300ms or after movement onset (25% of all active trials and 16% of all passive trials). Moreover, we excluded those conditions in which the *d'* was nearly zero or the relative *c* was larger than 30, both suggesting a failure to discriminate signal from noise. These corresponded to less than 1% (0.008) of all conditions.

EMG analysis was conducted with all the valid data available after the first exclusion criterion (see above). Data from one participant in the cue+ condition was excluded due to technical failure in the EMG recording. The following data were excluded due to extreme noise: one participant all blocks, one participant cue+ blocks, one participant one block in cue- condition. Finally, active cue- data from one participant was excluded as an extreme outlier.

### Quantification and statistical analysis

#### Statistical analysis

Statistical analyses at the group-level were implemented in R Studio version 2023 4.2.3.^89^ For all analyses, we implemented linear mixed models (LMMs) using the lmerTest package in R.^90^ The LMMs were fitted by the maximum likelihood estimation. For each signal detection measure (*d'*, relative *c*), the LMM included fixed effects terms of movement, cue and their interaction. As stimulus amplitude is known to influence tactile detection performance,^15^ we included amplitude as covariate of no interest. Participant was entered as a random effect term to account for within-subject variability. We checked the model assumptions with a simple model consisting of fixed and random effects (fixed effects of cue, movement, amplitude and random effect of participant) using the check_model function implemented in R.^91^ Visual inspection of model assumptions did not reveal any obvious deviations from normality of residuals, random effects, linear relationship, homogeneity of variance and multicollinearity. For all fixed effects of interest, we used dummy coding. We estimated the fixed effect terms with the anova function which performs an F test on the fixed effects using the Satterthwaite approximation.^92^ For post-hoc pairwise comparisons and estimated marginal means, we used the emmeans package.^93^ For multiple pairwise comparisons, we used Bonferroni adjustment, when necessary. In addition, we computed 95% confidence intervals for the fixed effect coefficients using the confint function. Effect sizes were computed using the effectsize package in R^94^ and reported as Cohen’s *d*.

Tactile suppression has been shown to depend on the time between stimulus and movement onset^10^^,^^17^^,^^26^^,^^62^ and can be modulated by movement speed.^68^^,^^70^^,^^95^ As we did not have a direct measure of movement speed, we used movement duration as an indirect measure of movement speed. In order to test potential differences in kinematic parameters across conditions, which might influence tactile suppression, we initially tested potential differences in these parameters as a function of condition, and if these parameters correlated with signal detection measures. For each condition of interest (movement, cue), we conducted LMMs with movement, cue and their interaction as fixed effects of interest and participant as a random effect. We conducted a similar LMM on the EMG data (mean EMG signal, see EMG preprocessing and analysis) with movement, cue and their interaction as fixed effects and participant as a random effect. For correlation analyses, we obtained Spearman correlation coefficients corresponding to the relationship between movement duration as well as stimulation onset relative to movement onset and signal detection measures. For determining the significance of correlations, we set the α = 0.05 while correcting for multiple correlations using the Bonferroni procedure (0.05 / 8 = 0.006 for each movement parameter).

## Supplemental information

Document S1. Figures S1–S7 and Tables S1–S3

Data S1. Participant demographic information
