# Supplementary material for: Distinct role of central predictive mechanisms in tactile suppression
Source: iScience. 2024 Jul 25;27(8):110582. doi: 10.1016/j.isci.2024.110582 (PMC11345528; doi:10.1016/j.isci.2024.110582)
Supplement: Document S1. Figures S1–S7 and Tables S1–S3 [file mmc1.pdf]

iScience, Volume *27*

Supplemental information

Distinct role of central predictive mechanisms in tactile suppression

Belkis Ezgi Arikan, Dimitris Voudouris, Benjamin Straube, and Katja Fiehler

Table S1. Fixed effects of interest from the LMM on *stimulation relative to movement onset* related to Figure 3C***.*** Statistics are reported for each item with sum of squares (*SS*), mean squares (*MS*), degrees of freedom (*dfs*), F-value (*F*) and p-value (*p*).

	SS	*MS*	*dfs*	*F*	*p*
Movement	5238.1	5238.1	1, 618.77	7.88	0.005
Cue	203.0	203.0	1, 644.26	0.30	0.58
Movement x cue	582.1	582.1	1, 618.83	0.88	0.35
Amplitude (covariate)	878.5	878.5	1, 618.08	1.32	0.25

Table S2. Fixed effects of interest from the LMM on *movement duration* related to Figure 3B***.*** Statistics are reported for each item with sum of squares (*SS*), mean squares (*MS*), degrees of freedom (*dfs*), F-value (*F*) and p-value (*p*).

	SS	*MS*	*dfs*	*F*	*p*
Movement	134999	134999	1, 617.72	53.35	< 0.001
Cue	558	558	1, 629.07	0.22	0.64
Movement x cue	1310	1310	1, 617.74	0.52	0.47
Amplitude (covariate)	640	640	1, 617.51	0.25	0.62

Table S3. Planned post-hoc pairwise comparisons on the *detection rate* as a function of movement and time bin related to Figure 2**.** Statistics are reported for each item with estimate, standard error (*SE*), degrees of freedom (*df*), t-value (*t*) and Bonferroni-corrected p-value (*p*).

Contrast	*Estimate*	*SE*	*Df*	*t*	*p*
Active bin 1 – passive bin 1	-0.10	0.04	586	-2.75	0.03
Active bin 2 – passive bin 2	-0.18	0.04	586	-5.16	< 0.001
Active bin 3 – passive bin 3	-0.15	0.04	586	-4.26	< 0.001
Active bin 4 – passive bin 4	-0.03	0.04	586	0.82	1
Active bin 5 – passive bin 5	0.02	0.04	586	0.55	1

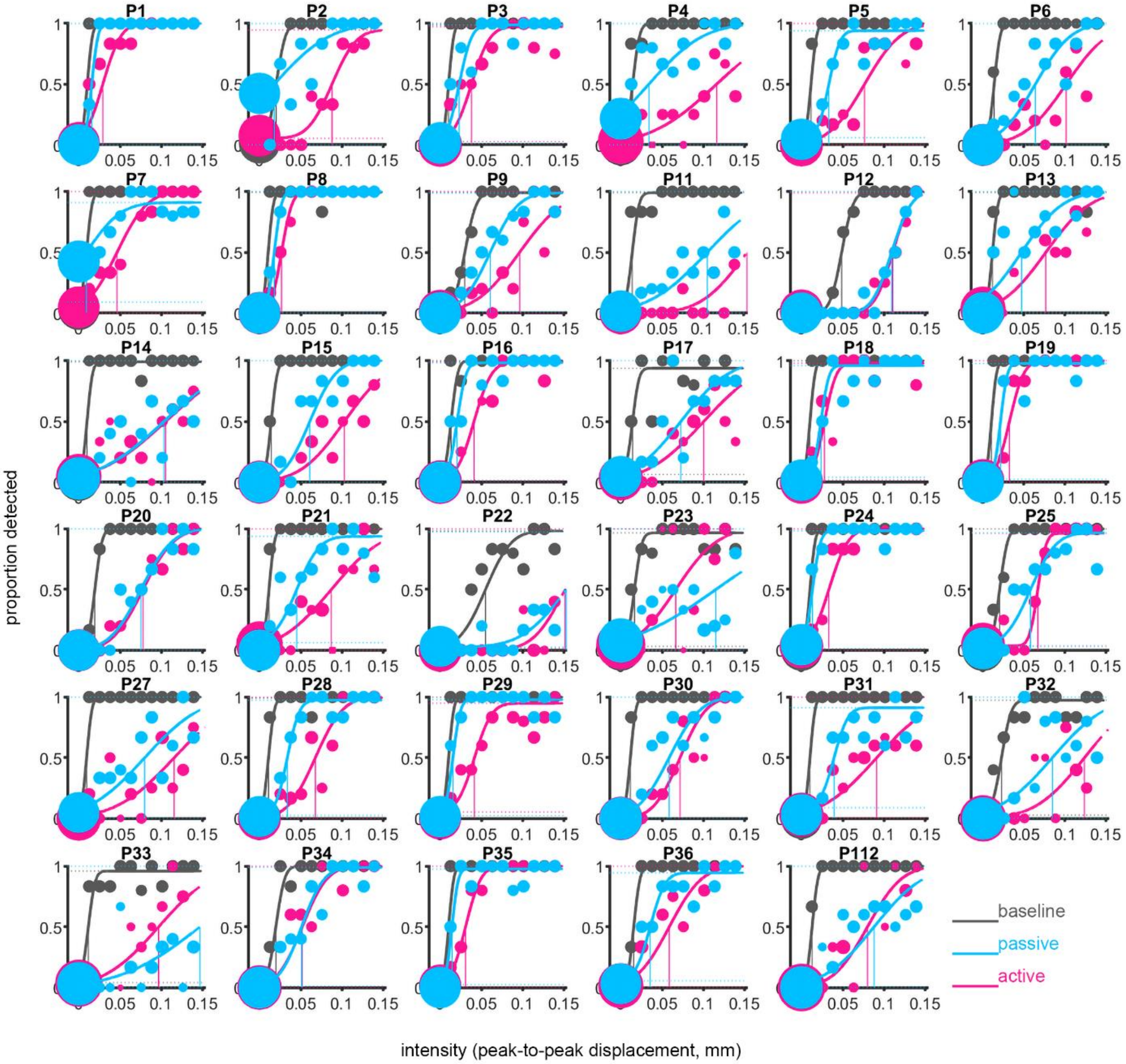

Figure S1. Individual psychometric functions depicting tactile detection as a function of vibrotactile probe intensity in the cue- condition related to Figure 1**.** The size of the symbols indicates the number of presented trials for each probe intensity. Horizontal lines indicate lapse (upper boundary) and guess (lower boundary) rates.

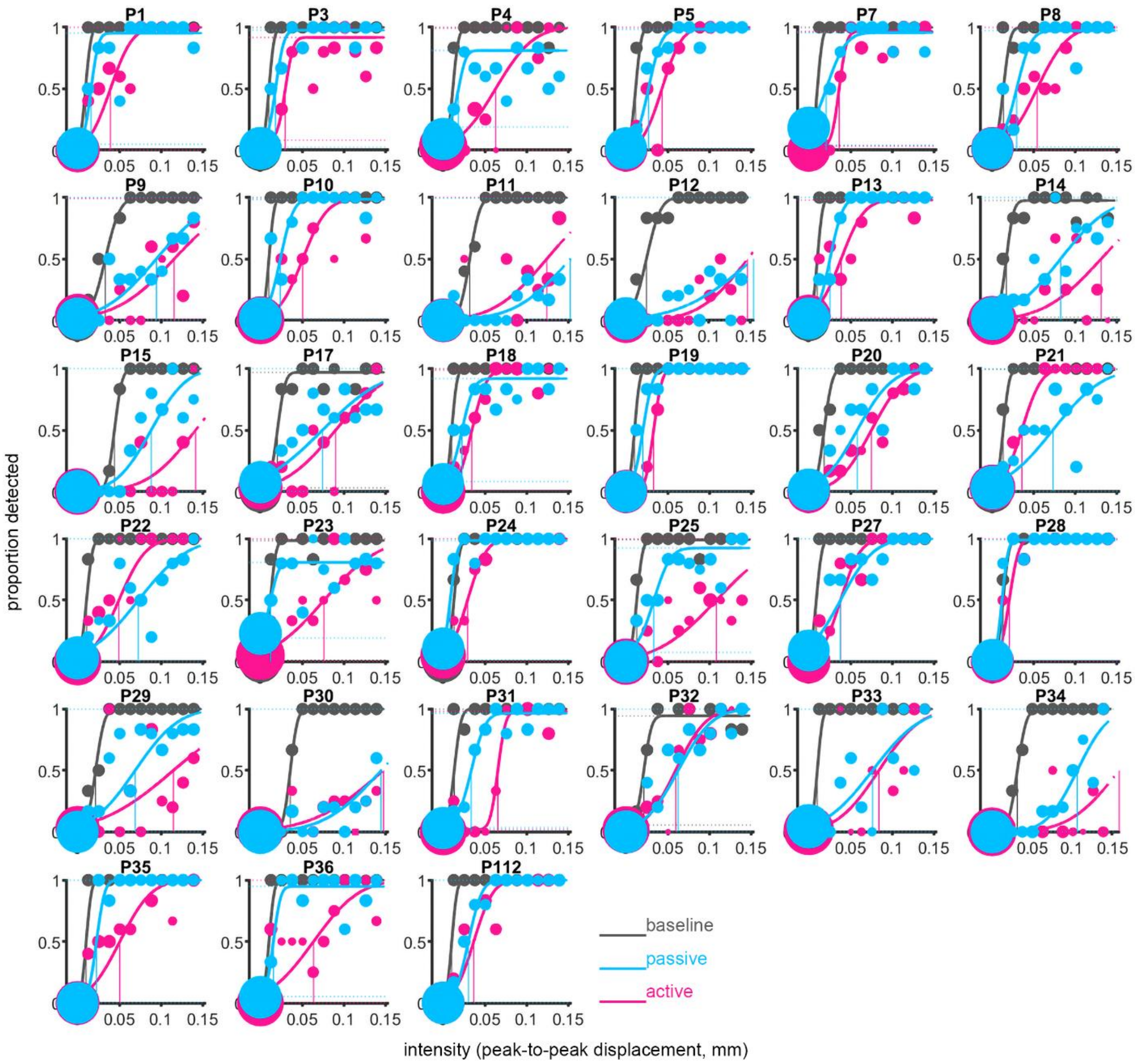

Figure S2. Individual psychometric functions depicting tactile detection as a function of vibrotactile probe intensity in the cue+ condition related to Figure 1**.** The size of the symbols indicates the number of presented trials for each probe intensity. Horizontal lines indicate lapse (upper boundary) and guess (lower boundary) rates.

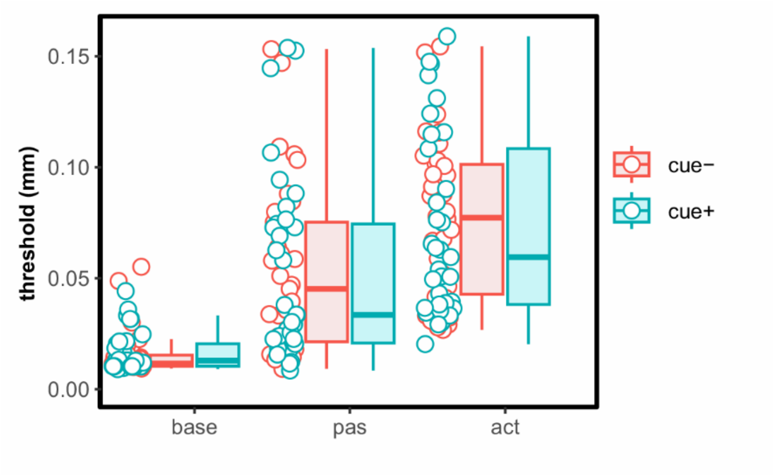

Figure S3. Group-level detection thresholds as a function of movement and cue related to Figure 1**.** Dots represent individual data points (n = 35 in cue- and n = 33 in cue+).

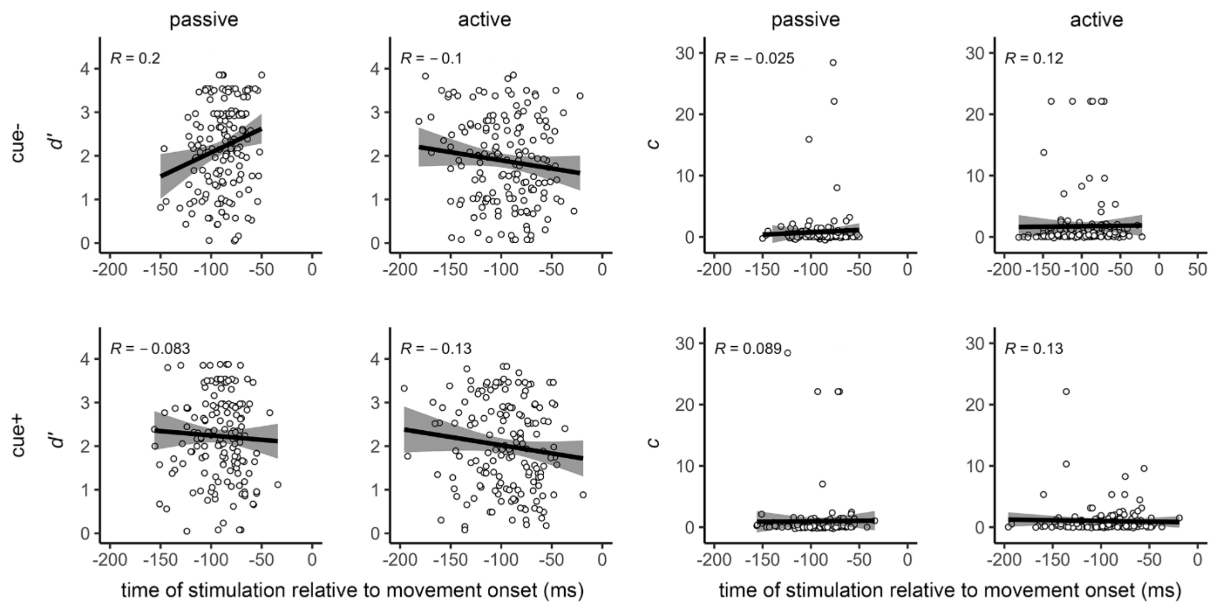

Figure S4. Correlation between detection measures of *d'* and *c*, and stimulus presentation relative to movement onset related to Figure 2 and Figure 3**.** Dots represent individual data points (n = 35 in cue- and n = 33 in cue+). Line and shading represent the best-fitting regression line and its 95% confidence interval, respectively. All *p* values > .05 after correction for multiple comparisons.

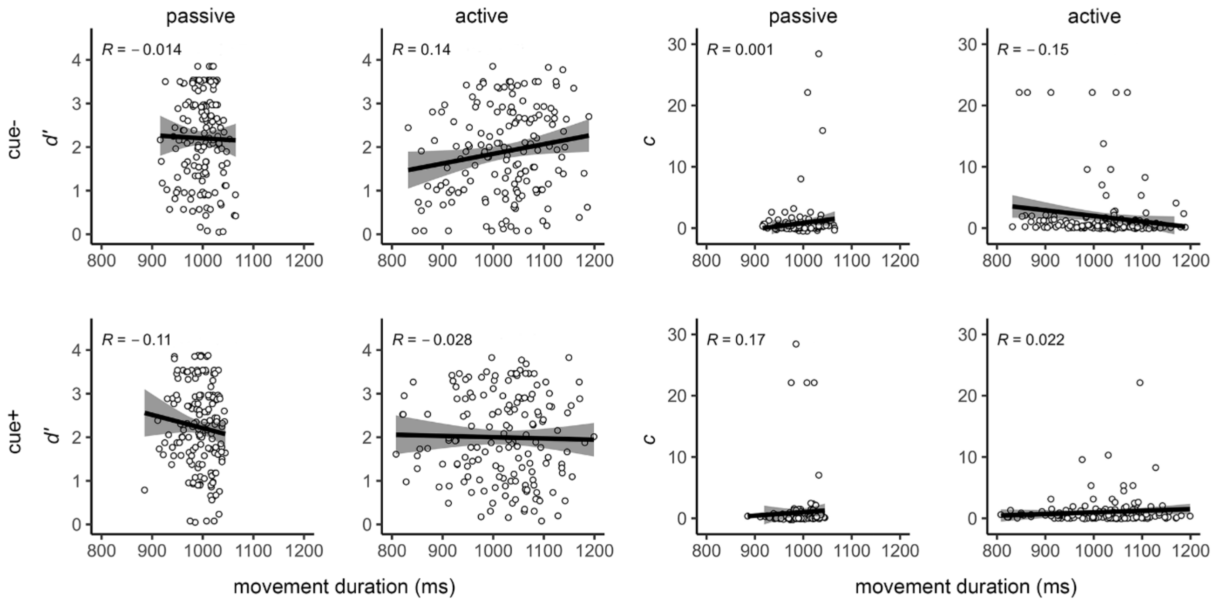

Figure S5. Correlation between detection measures of *d′* and *c*, and movement duration related to Figure 2 and Figure 3**.** Dots represent individual data points from all participants (n = 35 in cue- and n =33 in cue+). Line and shading represent the best-fitting regression line and its 95% confidence interval, respectively. All *p* values > .05 after correction for multiple comparisons.

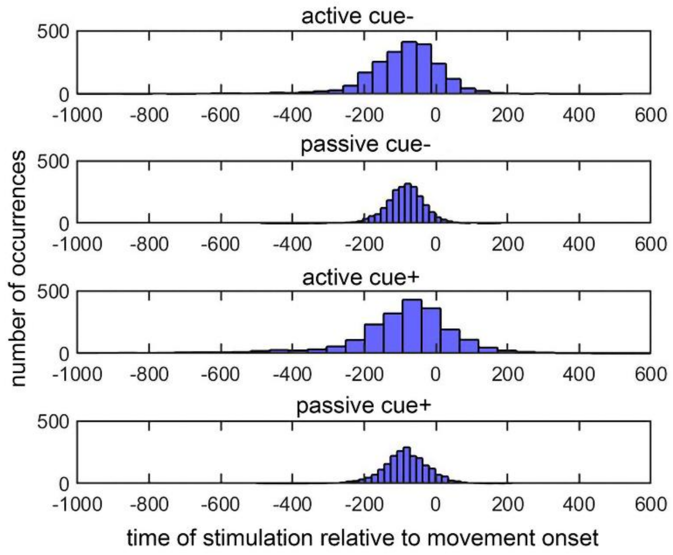

Figure S6. Distribution of stimulation onsets relative to movement onsets in each condition collapsed across all participants related to Figure 1**.** Negative values indicate stimulation occurring before movement.

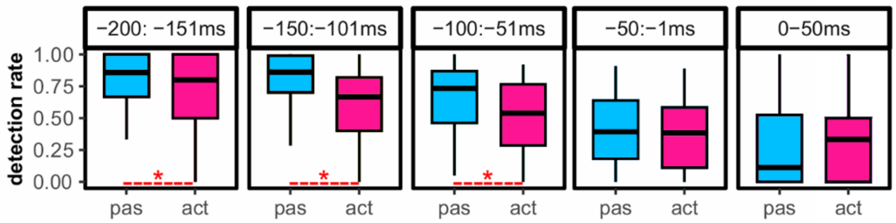

Figure S7. Time course of detection as a function of movement and cue related to Figure 2**.** In order to test if time course of tactile suppression differed between active and passive movements, we conducted an analysis on detection judgments, i.e. to trials in which a vibrotactile stimulus was present and the stimulation occurred max. 200ms before movement onset (-200ms) and max. 50ms (50ms) after movement onset based on the available data in each condition of interest (active, passive cued and uncued) (see also Figure S6 for a distribution of all trials in each condition of interest). For each participant and condition, we then binned the detection responses as a function of stimulation time relative to movement onset into five bins (-200:50:50ms) and calculated the proportion of detected trials in each bin. Data from one participant in the active cue+ condition was excluded due to 0 detection responses in all bins. In addition, we calculated the median stimulation amplitude in each bin in order to account for potential influence of overall vibration intensity on detection judgments.[1] To determine if time course of tactile sensitivity differed as a function of movement and time, we conducted a LMM with interaction between movement and time bin as fixed factors of interest along with movement, time bin, cue and their interaction as fixed factors, participant as random factor and amplitude (median amplitude in each bin) as covariate. The LMM showed an interaction between movement and time bin; $F(4, 586.17) = 5.51$, $p < .001$. For the interaction effect, planned post-hoc comparisons looking at detection rate in each bin as a function of movement revealed significant differences in the active compared to the passive condition in time bins 1 to 3, showing decreased detection rates within the 200-50ms time bin for active as opposed to passive movements. Significant differences revealed by the LMM analysis are indicated by bold asterisks (n = 35 in cue- and n = 32 in cue+).

Supplemental references

1. Williams, S.R., and Chapman, C.E. (2000). Time Course and Magnitude of Movement-Related Gating of Tactile Detection in Humans. II. Effects of Stimulus Intensity. J. Neurophysiol. *84*, 863–875. https://doi.org/10.1152/jn.2000.84.2.863.
